# Time versus energy minimization migration strategy varies with body size and season in long-distance migratory shorebirds

**DOI:** 10.1186/s40462-017-0114-0

**Published:** 2017-11-07

**Authors:** Meijuan Zhao, Maureen Christie, Jonathan Coleman, Chris Hassell, Ken Gosbell, Simeon Lisovski, Clive Minton, Marcel Klaassen

**Affiliations:** 1 Centre for Integrative Ecology, School of Life and Environmental Sciences, Deakin University, Geelong, Australia; 2 Victorian Wader Study Group, c/o 165 Dalgetty Road, Beaumaris, Vic 3193 Australia; 3 Queensland Wader Study Group, 22 Parker Street, Shailer Park, Qld 4128 Australia; 4 Global Flyway Network, PO box 3089, Broome, Australia; 5 Schweizerische Vogelwarte, Department of Bird Migration, 1 Seerose, 6204 Sempach, Switzerland

**Keywords:** Optimal migration theory, Migration strategy, Time-minimization, Energy-minimization, Light-level geolocator, Migration speed, Body size

## Abstract

**Background:**

Migrants have been hypothesised to use different migration strategies between seasons: a time-minimization strategy during their pre-breeding migration towards the breeding grounds and an energy-minimization strategy during their post-breeding migration towards the wintering grounds. Besides season, we propose body size as a key factor in shaping migratory behaviour. Specifically, given that body size is expected to correlate negatively with maximum migration speed and that large birds tend to use more time to complete their annual life-history events (such as moult, breeding and migration), we hypothesise that large-sized species are time stressed all year round. Consequently, large birds are not only likely to adopt a time-minimization strategy during pre-breeding migration, but also during post-breeding migration, to guarantee a timely arrival at both the non-breeding (i.e. wintering) and breeding grounds.

**Methods:**

We tested this idea using individual tracks across six long-distance migratory shorebird species (family Scolopacidae) along the East Asian-Australasian Flyway varying in size from 50 g to 750 g lean body mass. Migration performance was compared between pre- and post-breeding migration using four quantifiable migratory behaviours that serve to distinguish between a time- and energy-minimization strategy, including migration speed, number of staging sites, total migration distance and step length from one site to the next.

**Results:**

During pre- and post-breeding migration, the shorebirds generally covered similar distances, but they tended to migrate faster, used fewer staging sites, and tended to use longer step lengths during pre-breeding migration. These seasonal differences are consistent with the prediction that a time-minimization strategy is used during pre-breeding migration, whereas an energy-minimization strategy is used during post-breeding migration. However, there was also a tendency for the seasonal difference in migration speed to progressively disappear with an increase in body size, supporting our hypothesis that larger species tend to use time-minimization strategies during both pre- and post-breeding migration.

**Conclusions:**

Our study highlights that body size plays an important role in shaping migratory behaviour. Larger migratory bird species are potentially time constrained during not only the pre- but also the post-breeding migration. Conservation of their habitats during both seasons may thus be crucial for averting further population declines.

**Electronic supplementary material:**

The online version of this article (10.1186/s40462-017-0114-0) contains supplementary material, which is available to authorized users.

## Background

Long-distance migration has evolved independently in multiple taxa enabling animals to exploit spatially and temporally discrete peaks in resources [1], ultimately allowing increased reproductive output and survival [2]. However, migration is risky and energetically costly, and selection for optimal migratory strategies is paramount to ensure the benefits of migration outweigh the risks [3]. These considerations have led to the formulation of optimal migration theory and notably the time- and energy-minimization hypotheses [3]. The time-minimization hypothesis assumes that animals migrate at their maximum speed and thus complete their migration as fast as possible given constraints on flying speed and fuel deposition rate. The energy-minimization hypothesis assumes that migrants use the minimum amount of energy by either minimizing energy cost of transport per unit distance or by minimizing total energy cost of migration [4]. We refer to these as energy-minimization strategy without differentiating between the two. Accordingly, both the time and energy minimization hypotheses yield considerably different predictions for how migratory birds move in space and time during their annual migrations.

Optimal migration theory suggests that animals may adopt a time- or energy-minimization strategy depending on the season of migration [3, 5]. Time-minimization is commonly thought to play a major role during pre-breeding migration from the non-breeding (i.e. wintering) grounds to the breeding grounds [4], where a timely arrival provides a competitive advantage [6, 7] and guarantees optimal use of seasonally available local resources [8, 9], benefiting reproductive performance [10, 11]. Conversely, post-breeding migration is expected to be less time constrained [12], because it is generally assumed that a timely arrival at the wintering grounds has fewer fitness consequences [13]. Migrants are therefore expected to use an energy-minimization strategy during post-breeding migration [4].

Individuals employing a time- or an energy-minimization strategy are expected to differ in a range of quantifiable migratory behaviours [3, 4, 14, 15] (Table 1). Time-minimizers should take less time to complete their migration and therefore fly and migrate faster (i.e. minimize time spent in both flight and in preparing for migration) than energy-minimizers. In contrast, energy-minimizers are not constrained by time but limit energy use and therewith also require reduced foraging effort to deposit fuel and reduced fuel loads (e.g. [16]). Moreover, they may be more inclined to wait for favourable migration conditions to further reduce energy costs [17]. Habitat quality varies across sites and seasons, time-minimizers are proposed to be more selective in their habitat use, choosing high quality habitats allowing higher fuel deposition rates and shortened staging periods, ultimately promoting a faster migration [18]. Time-minimizers may thus bypass low quality sites and only stop at high quality sites. Energy-minimizers, on the other hand, should stop more regularly and deposit less fuel to avoid high fuel loads that are costly to carry and fly with [15]. Thus, time-minimizers are expected to take fewer and on average longer steps in completing their migration than energy-minimizers. Finally, time-minimizers might take longer routes than energy-minimizers, making detours via higher quality sites away from the direct migration route to speed up overall migration, even when this comes at higher flight costs [15, 19, 20]; obviously, time-minimizers do not have to migrate extra distance if quality sites are located along the direct migration route. Thus, we expect a similar or longer migration distance in time- compared to energy-minimizers.

**Table 1:** Expectation for the four migratory behaviours when migrants adopt a time- versus energy-minimization strategy

	Time-minimization	Energy-minimization
(*i*) Migration speed	high	low
(*ii*) Number of staging sites	few	many
(*iii*) Total migration distance	long or similar	short or similar
(*iv*) Maximum step length	long	short

To test the hypothesis that migrants adopt a time-minimization strategy during pre-breeding and an energy-minimization strategy during post-breeding migration, Nilsson C*,* et al. [21] reviewed studies and investigated multiple behaviours between seasons. They found supportive evidence, with faster average migration speeds, higher flying speeds and shorter migration durations during pre- compared to post-breeding migration. However, a number of studies in this review, together with other case studies (e.g. [22–24]), failed to detect a seasonal difference, or found even higher migration speeds during post-breeding migration [25, 26]. A range of explanations for these deviations from theory have been proposed. For example, Raess M [22] largely attributed this to the harsh environment, i.e. low temperature and poor vegetation availability that Siberian Stonechats (*Saxicola torquata maura*) encountered during their pre-breeding migration, delaying their arrival and lowering their average speed of migration. In another example involving Bewick’s Swan (*Cygnus columbianus bewickii*), Nuijten RJM*,* et al. [25], attributed the faster post-breeding than pre-breeding migration to either swans’ tendency to avoid being trapped by ice later in the season or swans potentially being capital breeders spending extra time depositing energy stores during their pre-breeding migration. Importantly, these studies do not consider the role of body size—which places physical constraints on flying speed [27–29], fuel deposition [30, 31], migration speed [32–34], migration distance [35, 36] and other life-history traits [33]—in determining the extent to which migrants adopt a time- or energy- minimization strategy, and how this may differ between pre- and post-breeding migrations.

Body size is an important determinant of the energy costs and speed at which life processes take place [37, 38]. The major life history events, i.e. breeding, moult and migration, generally take more time in large compared to small bird species [33] and it has consequently been argued that large migrants are potentially more time constrained than small birds [39]. Accordingly, large migratory birds may not only be time constrained during pre-breeding, but also during post-breeding migration. For instance, large-sized migrants may need to arrive at the wintering grounds sooner so that they can start moulting sooner and thus complete their moulting before the onset of the premigratory stage. If this is the case for relatively large migrants, then it may be expected that their migratory behaviours may not differ significantly between pre-breeding and post-breeding migrations.

In this study, we tested two hypotheses. First, we tested the hypothesis that birds minimize time use on their pre-breeding migration, while they minimize energy use on post-breeding migration. We accordingly expect that pre-breeding migrations are associated with 1) faster migration speed 2) smaller number of staging sites 3) potentially longer migration distance and 4) longer step length. We should bear in mind that these are largely non-distinctive characteristics of a time-minimization strategy. For instance, a faster pre-breeding migration speed could also be caused by more favourable environmental conditions for migration rather than higher time constraints during the pre- compared to post-breeding period (e.g. longer days for foraging, higher food availability, more prevailing tailwind). Nevertheless, examining these four migration variables in concert, should provide us with a good proxy of the migration strategies employed during both migration seasons. Next, we tested whether any seasonal difference in migratory behaviour decreases with body size.

To this end, we used detailed individual tracks obtained from light-level geolocation (‘geolocators’). To reduce the potential interference from phylogeny and ecology, we limited our study to six species of closely related (family Scolopacidae within the order Charadriiformes), long-distance (8000–13,000 km) migratory shorebirds using a single flyway (East Asian-Australasian Flyway, hereafter EAAF) across a large size range (50–750 g in lean body mass).

## Methods

We obtained complete, individual tracking data from six shorebird species: Sanderling (*Calidris alba*)*,* Ruddy Turnstone (*Arenaria interpres*) (hereafter Turnstone), Grey-tailed Tattler (*Tringa brevipes*) (Tattler), Red Knot (*Calidris canutus*), Great Knot (*Calidris tenuirostris*), and Far-eastern Curlew (*Numenius madagascariensis*) (Curlew). All species migrate between their wintering grounds in Australia and New Zealand, and their breeding grounds in northeast China and Siberia. Individual tracking data were either not published or were extracted from publications, detailed information on species and tracking data compilation can be found in Table 2. For each individual, data for pre- and post-breeding migration was collected from successive seasons, mostly in the same year. But individuals within or across species were tracked across years between 2009 and 2014 (Table 2). Method of geolocator data processing, from light-level to estimation of spatial-temporal data, followed Lisovski S*,* et al. [40] for Sanderling, Turnstone and Great Knot. BASTrack software was used to process data for other species, with further details and the methods used being provided in the respective publications (Table 2).

**Table 2:** Details of the six EAAF migratory sandpipers that were tracked using geolocators

Species	Lean body mass (g)^a^	Wintering site	Breeding grounds	n	Year
Sanderling *Calidris alba*	50^b^	South Australia	Arctic Russia	12^c^	2012
Ruddy Turnstone *Arenaria interpres*	93^b^	Tasmania, Victoria, South Australia	Arctic Russia	60^d^	2009–2014
Grey-tailed Tattler *Tringa brevipes*	108^e^	Queensland	Russia’s far east	3^f^	2011
Red Knot *Calidris canutus*	113^g^	New Zealand	Arctic Russia	2^h^	2011–2012
Great Knot *Calidris tenuirostris*	135^b^	North Western Australia	Arctic Russia	7^i^	2013
Far Eastern Curlew *Numenius madagascariensis*	743^b^	Victoria	sub-Arctic between northeast China and Russia	9^j^	2011–2012

^a^lean body mass obtained from the literature or ^b^calculated as the median body mass of individuals captured during November–December on their wintering grounds in Australia (unpubl. Data Victorian Wader Study Group and Australasian Wader Studies Group) and multiplied by 0.94 [41]. ^c^Tracks were extracted from Lisovski S*,* et al. [40]. ^d^Unpubl. tracks from Victorian Wader Study Group, Australia. ^e^Tracks were extracted from Johnsgard PA [76]. ^f^Unpubl. tracks from Queensland Wader Study Group, Australia. ^g^Tracks were extracted from Battley PF [77]. ^h^Tracks were extracted from Tomkovich PS*,* et al. [78]. ^i^Tracks were extracted from Lisovski S*,* et al. [68]. ^j^Unpubl. tracks from Victorian Wader Study Group, Australia, and extracted tracks from Minton C and Gosbell K [79]

For body size we used lean body mass (g), i.e. body mass of a bird without any migratory fuel, obtained from the literature or, if unavailable, estimated as the median body mass of individuals captured during November–December in Australia (unpublished data, Victorian Wader Study Group and Australasian Wader Studies Group) and multiplying this by 0.94 following [41].

Four migration variables depicting aspects of the migratory itineraries were extracted from the tracks for each individual tracked for both pre-breeding and post-breeding migration separately, namely (*i*) migration speed, (*ii*) number of staging sites, (*iii*) total migration distance, and (*iv*) maximum step length. Migration speed (*i*) was calculated by dividing the total migration distance by the total migration duration. As migratory birds at the start of their migration (i.e. wintering grounds prior to pre-breeding migration and breeding grounds prior to post-breeding migration) commence fuelling before departure on their first migratory leg, this “premigratory” staging period should be taken as part of migration [3]. This premigratory staging period, however, was impossible to ascertain from the tracking data. To ensure that any pattern we might detect in migration speed with size was not caused by our subjective selection of methods, we endeavoured to use two methods to calculate migration speed, naming them as *traditional* migration speed and *partial* migration speed. In brief, *traditional* migration speed was calculated by dividing total migration distance by total migration duration. We defined total migration duration as the days elapsed from the date on which the birds left their wintering/breeding grounds to the date on which the birds arrived on their breeding/wintering grounds. *Partial* migration speed was the migration speed excluding the first migratory leg, and was calculated by dividing migration distance between the first staging site and the wintering/breeding location by the time elapsed from the date of arrival at the first staging site and the date of arrival at the wintering/breeding grounds. Although *partial* migration is probably the less biased estimate of an individual’s migration speed, we consider that collectively the two estimates provide a better insight in migration speed variations among the focal species.

We defined “true” staging sites as sites where birds stopped for a duration of at least 4 days (see [42] for definition of staging and stopover site) and the number of staging sites (*ii*) was calculated accordingly. A migratory leg is considered to be the journey connecting the subsequent “true” staging, wintering or breeding site. Step length (*iv*) was the distance travelled in one migratory leg via a great circle route. Due to the limited resolution in geographic position using geolocators, the step length estimation for some short steps may be associated with high errors. To avoid this problem, we used the maximum step length during each migration to represent step length. Maximum step length rather than mean or median step length was also considered for other reasons. During pre-breeding migration, a few shorebird species fly non-stop for over 5000 km from Australia and New Zealand to the East Asian coast [43, 44], bypassing potential stopover sites on the Pacific Islands. Thereafter, they move along the East Asian coast with small steps before flying to their breeding grounds (Far Eastern Curlew as an exception). Given the high food availability across the area [43], time-minimizers are likely to stop frequently to fuel up at all possible stopover sites in East Asia, and avoid to carry and fly with high fuel loads [15], thus behaving similarly to energy-minimizers. This contrasts with scenarios where high quality sites are only sparsely distributed and time-minimizers have to deposit high fuel loads to make it between high quality sites [15]. To distinguish between time- and energy-minimization, maximum step length compared to the average or median step length therefore serves as a better proxy. A longer maximum step would be observed if migrants fly non-stop between Australia/New Zealand and East Asia, as opposed to stopping in the tropical islands in between. Total migration distance (*iii*) was the sum of each step length (Additional file 1: Table S1 in provides data of seasonal difference, i.e. pre- minus post-breeding migration, for all four investigated migration variables for each individual).

### Statistical analysis

To compare the seasonal difference between pre- and post-breeding migration for each of the migration variables (including two estimates of migration speed, number of staging site, total migration distance and maximum step length), we used two-tailed paired t-tests for Turnstone and, because of non-normality of the data, Wilcoxon signed rank test for Sanderling, Great Knot and Curlew. For Tattler and Red Knot where sample size was too small (2 and 3 respectively), seasonal difference was described but no statistical test was applied. To further examine if a seasonal difference existed across species, analysis of variance (ANOVA) was used. Before performing ANOVA, we firstly checked homogeneity of variances across species using Bartlett test. One-way ANOVA was used if homogeneity of variances held true, otherwise Welch’s ANOVA was used to account for heterogeneity; both types of ANOVA were followed by a post-hoc pairwise multiple comparison test. Considering the unequal sample size and heterogeneity in most cases, we performed Games-Howell post-hoc tests using the ‘posthocTGH’ function in the R-package ‘userfriendlyscience’.

To examine if the between-species difference in a migration variable varied in a size related manner, we compared three linear model structures. All three linear models included the seasonal difference as the response variable and size as a covariate, but with different explanatory structures: 1) general linear model, including size as the only fixed variable; 2) general linear mixed model, besides including size as the only fixed variable, fitting species as a random factor; and 3) general linear mixed model, besides including size as the only fixed variable and fitting species as a random factor, also accounting for potential heterogeneity of variances between species. The three models were compared and the one with the lowest Akaike information criterion was selected as the final model structure. Using the final structure, we compared models with and without size as a covariate and displayed the ΔAIC between the two. This procedure was repeated for each migration variable for which ANOVA/Welch’s ANOVA revealed a between-species difference. We used the 10-log of lean body mass to normalise the body size data. We conducted the linear mixed models using the ‘lme’ function in R-package ‘nlme’. Accounting for heterogeneity of variances was done by specifying there is heterogeneity between species using the ‘varIdent’ function [45].

Although we limited our studied species to six closely related species within a single family to largely avoid potential phylogeny interference, phylogenetic differences still exist. To examine the potential interference of phylogeny, we used a phylogenetic mixed model to examine the effect of size on all migration variables that showed a between species difference. To this end, we used a Bayesian approach, applying the ‘MCMCglmm’ function within the R-package ‘MCMCglmm’. Data were transformed prior to analyses as outlined above for linear models. We defined the data distribution as Gaussian for all migration variables. We used a phylogenetic tree based on data provided in Thomas GH*,* et al. [46]. All analyses were conducted using R version 3.2.3 [47].

## Results

### Migration speed

**Fig. 1:**
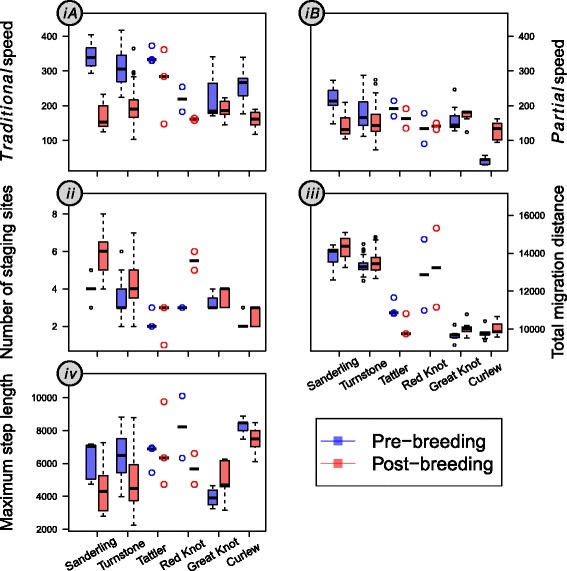
Four migratory variables for pre- and post-breeding migration in six EAAF migratory sandpipers. (***iA***) *traditional* migration speed (km/d) calculated as the total migration distance divided by total migration duration; (***iB***) *partial* migration speed (km/d), dividing migration distance between the first staging site and the wintering/breeding grounds by the total duration from arrival at the first staging site until arrival at the breeding/wintering grounds; (***ii***) number of staging sites; (***iii***) total migration distance (km); (***iv***) the maximum step length (km). Species along the X-axes are ranked in order of increasing lean body mass. The thick line within each box and whisker plot represents the median, and the lower and upper box border represents the first and the third quartile, respectively. Whiskers denote the lower and upper 95% confidence interval. Dots outside the whiskers are outliners above or below the 95% confidence interval. Because of low samples size in Tattler and Red Knot (i.e. three and two data points, respectively) individual data points and medians are presented for these species

**Fig. 2:**
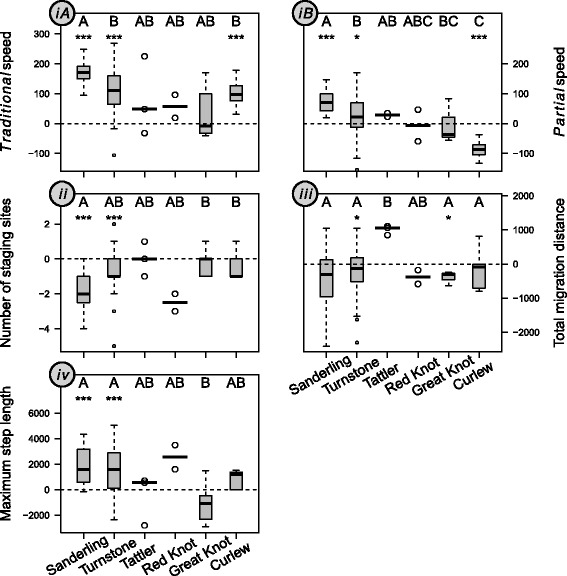
Seasonal difference in four migratory variables for six EAAF migratory sandpipers. Seasonal difference was based on subtracting data for post-breeding from pre-breeding migration for each individual bird. (***iA***) *traditional* migration speed (km/d) calculated as the total migration distance divided by total migration duration; (***iB***) *partial* migration speed (km/d), dividing migration distance between the first staging site and the wintering/breeding grounds by the total duration from arrival at the first staging site until arrival at the wintering/breeding grounds; (***ii***) number of staging sites; (***iii***) total migration distance (km); (***iv***) the maximum step length (km). Species along the X-axes are ranked in order of increasing lean body mass. The thick line within each box and whisker plot represents the median, and the lower and upper box border represents the first and the third quantile, respectively. Whiskers denote the lower and upper 95% confidence interval. Dots outside the whiskers are outliners above or below the 95% confidence interval. Since only three and two data points were available for Tattler and Red Knot, respectively, the individual data points and the medians are plotted for each of these two species. In all panels a dotted horizontal line representing no seasonal difference is added to assist visual interpretation. For example, in panel (***iA***) below the zero horizontal line indicates slower pre- compared to post-breeding migration speed, whereas above the line indicates faster pre- compared to post-breeding migration. Seasonal difference within species differences were tested using paired t-tests asterisks identifying significant differences (*p* < 0.05 ‘*’, *p* < 0.01 ‘**’, *p* < 0.001 ‘***’). Between species differences were tested using multiple comparison Games-Howell post-hoc tests and are noted with capital letters, with species not sharing the same letter being significantly different. Liner models showed that only *partial* migration speed (***iB***) varies in relation to lean body mass, with post-breeding migration becoming progressively faster relative to pre-breeding migration with an increase in body size

For both *partial* and *traditional* migration speed most species migrated at similar or faster speed during pre- compared to post-breeding migration (Fig. 1), supporting the hypothesis that migrants generally are time-minimizers during pre-breeding migration. The faster pre- compared to post-breeding migration speed was significant and most pronounced in the smallest sized species, Sanderling and Turnstone (Table 3; Fig. 2 *iA, iB*). No (apparent) differences between seasons were detected in the three medium-sized species Great Knot (Table 3, Fig. 2 *ib, iB*) and Tattler and Red Knot (no statistical tests applied due to small sample size; Fig. 2 *iA, iB*). The seasonal difference was also significant in the largest sized species, Curlew, but the direction of the seasonal difference was opposing with a faster pre-breeding *traditional* migration speed (pre-breeding minus post-breeding: 96 km /d, Fig. 2*iA*) and a slower pre-breeding *partial migration speed* (−87 km/d, Fig. 2*iB*). Collectively, the species-specific differences between pre- and post-breeding migration speeds resulted in a significant decline in the difference in *partial* migration speed between the two seasons with size (Fig. 2 *iB* and Additional file 2: Fig. S1), supporting our prediction. This was revealed by both the model correcting for phylogeny (slope = −143, *p* = 0.020) and the model without correcting for phylogeny (slope = −135, t_89_ = −5.22, *p* < 0.001), with AIC being much lower for the model with than without size as a covariate (Table 4). Although seasonal difference in *traditional* migration speed showed a very weak tendency in the same direction, this was far from significant (*traditional* speed correcting for phylogeny: slope = −96, *p* = 0.220; *not* correcting for phylogeny: slope = −44, t_6,93_ = −0.79, *p* = 0.470), despite the inclusion of size leading to a considerably better fitting model (Table 4).

**Table 3:** Results of seasonal difference between pre- and post-breeding migration in four EAAF migratory sandpipers

	Sanderling	Turnstone	Great Knot	Curlew
n	12	60	7	9
(*i*) Migration speed				
*Traditional*	**V = 78 (** ***p*** **= 0.000)**	**t**_**59**_ **= 12.2 (** ***p*** **= 0.00)**	V = 17 (*p* = 0.688)	**V = 45 (** ***p*** **= 0.004)**
*Partial*	**V = 78 (** ***p*** **= 0.000)**	**t**_**59**_ **= 2.7 (** ***p*** **= 0.010)**	V = 10 (*p* = 0.578)	**V = 0 (** ***p*** **= 0.031)** ^**a**^
(*ii*) Number of staging sites	**V = 0 (** ***p*** **= 0.003)**	**t**_**59**_ **= −4.9 (** ***p*** **= 0.000)**	V = 2.5 (*p* = 0.424)	V = 3.5 (*p* = 0.129)
(*iii*) Total migration distance	V = 20 (*p* = 0.151)	**t**_**59**_ **= −2.44 (** ***p*** **= 0.018)**	**V = 0 (** ***p*** **= 0.016)**	V = 8 (*p* = 0.353)
(*iv*) Maximum step length	**V = 75 (** ***p*** **= 0.002)**	**t**_**59**_ **= 6.23 (** ***p*** **= 0.00)**	V = 4 (*p* = 0.109)	V = 15 (*p* = 0.059)

^a^For three Curlews migrating around the equinox their timing of migration and there with their partial migration speed could not be determined with sufficient accuracy, resulting in a sample size of 6 instead of 9 for this variable

Paired-t test was used for Turnstone and Wilcoxon signed rank test was used for the other three species. n depicts sample size. Significant results (*p* < 0.05) are highlighted in bold

**Table 4:** Comparisons of linear model performances of the migration variables with and without size as a covariate in terms of difference in AIC values (ΔAIC)

	Explanatory variables	ΔAIC
(*i*) Migration speed		
*Traditional*	~ 1	8.5
	~ 1+ size	0
*Partial*	~ 1	21.3
	~ 1+ size	0
(*ii*) Number of staging sites	~ 1	1.0
	~ 1+ size	0
(*iii*) Total migration distance	~ 1	10.6
	~ size	0
(*iv*) Maximum step length	~ 1	14.7
	~ 1+ size	0

In all cases a ΔAIC of 0 defines the best model. ΔAIC of alternative models that are >2 suggest that these alternative models are performing worse than the model with a ΔAIC equalling 0

### Number of staging sites

All species made either fewer or a similar number of stops during pre- compared to post-breeding migration. Although weak in support only, this observation is consistent with the prediction that migrants minimize time use when migrating towards their breeding grounds (Fig. 1 *ii*). Tattler, Red Knot (Fig. 2 *ii*), Great Knot and Curlew (Table 3 and Fig. 2 *ii*) used a similar number of staging sites during pre-breeding and post-breeding migration. The two small species, i.e. Sanderling and Turnstone, used more staging sites (Table 3 and Fig. 2 *ii*) during post-breeding migration. This seasonal difference was more pronounced in Sanderling (on average two more sites during post-breeding migration) compared to Turnstone (one more site). The seasonal difference in number of staging sites did not systematically vary with size, AIC value being similar between models including and not including size as a covariate (Table 4).

### Total migration distance

For the four species that were statistically tested, Sanderling and Curlew covered a similar total distance during post-breeding migration compared to pre-breeding migration (Table 3, Fig. 1 *iii*). The other two species Turnstone and Great Knot covered slightly longer distances during post- compared to pre-breeding migration). However, the seasonal difference was minimal (100 km and 300 km respectively), falling within the 200–400 km error range for geolocator data. Although untested, seasonal difference in total migration distance for Red knot was also small (380 km) and within the error range of geolocator data. Tattler was the only species taking a shorter post-breeding route by 1000 km (Fig. 2 *iii*). The overall similar or longer migration distance during pre- compared to post-breeding migration is in line with our expectation that migrants minimize time use and potentially choose a longer but faster route when migrating to the breeding grounds. The seasonal difference in total migration distance failed to show a size dependent pattern, despite that the model including size as a covariate performed better in terms of a lower AIC value compared to the model not including size (Table 4).

### Maximum step length

Maximum step length was similar or longer during pre- compared to post-breeding migration (Table 3, Fig. 1 *iv*), consistent with the prediction that migrants might make longer steps to minimize time use when migrating towards their breeding grounds. During post-breeding migration, all four statistically tested species took shorter (Sanderling, Turnstone and Curlew) or similar step length (Great Knot) (Table 3, Fig. 2 *iv*). Although statistical testing was not opportune given low sample sizes, Tattler and Red Knot also had similar or longer step lengths during pre- compared to post-breeding migration (Fig. 2 *iv*). The seasonal difference in maximum step length was significantly higher in Sanderling and Turnstone than Great Knot. We detect no size-related pattern in maximum step length, despite that model including size as a covariate performed better in terms of a lower AIC value compared to the model not including size (Table 4).

## Discussion

Using individual tracking data across six differently sized shorebird species migrating along the EAAF, we found support for the hypothesis that, overall and more often than not, migrants showed evidence of using a time-minimization strategy during pre-breeding migration and an energy-minimization strategy during post-breeding migration. Most species displayed one or more of the four seasonal differences, including migrating faster, using fewer staging sites, covering similar or longer total distance and making longer steps during pre- compared to post-breeding migration. Seasonal difference in the number of staging sites, total migration distance and maximum step length did not show any size-related pattern. Remarkably, we found that the seasonal difference in migration speed, the ultimate indicator of time- versus energy- minimization, tended to decrease with body size. Across seasons, larger species showed greater similarity in their migratory behaviour than small species. Assuming they were using a time-minimisation strategy during pre-breeding migration (for which e.g. a long maximum step length and few staging sites [Fig. 1] are indicative), this conforms to our additional hypothesis that large species are potentially more time constrained year around. Large species thus potentially not only adopt a time-minimization strategy during pre-breeding migration, but also during post-breeding migration. We acknowledge that migration speed was mainly represented as *partial* migration speed in this study; migration speed across the entire migration would be ideal and such study is warranted to further test our hypothesis. We also acknowledge that the four investigated migration variables can only suggest and not distinctively identify the use of a time- versus an energy- minimization strategy. There is still the probability that differences in environmental conditions rather than migration strategy underlie the seasonal differences observed in migratory behaviour. Nevertheless, evidence supporting the hypothesis was strong in terms of all four variables performing as expected. This is, to our knowledge, also the first study proposing and demonstrating that body size plays a key role in shaping migratory behaviours between seasons. This was evident in all but one (staging site) AIC tests for the effect of size (Table 4). Also, the potentially more conservative hypothesis-testing (i.e. *P* value) approach (see [48] for discussion), which we will preferentially refer to in our discussion below, indicated that size affected many of the migratory behaviours.

### Migration speed

The overall similar or higher migration speed during pre- compared to post-breeding migration is consistent with the findings in the review by Nilsson C*,* et al. [21]. Seasonal difference in *partial* migration speed declined significantly with size. The traditional migration speed tended to decline, although not significantly, corroborating the *partial* migration speed results of a decline in migration speed with size. This lack of seasonal difference in large species suggests that large compared to small species behave more like time-minimizers during both pre- and post-breeding migration. Alternatively, one might argue that large species might use energy- rather than time-minimization during both pre- and post-breeding migration. This, however, is unlikely to be the case due to the apparent high time constraints throughout the annual life cycle for large species and in these large species clear hallmarks of a time-minimisation strategy (e.g. small number of staging sites and long maximum step lengths, Fig. 1).

Besides migration and breeding, primary moult is also energy-costly [49]. Many long-distance migratory birds schedule these events so as to avoid overlap (e.g. passerines [50], shorebirds [51]). All six shorebird species in this study conduct most if not all of their moult on the wintering grounds [52]. Large species however tend to take more time to complete primary moult; e.g. moult takes an average of 98 days for the smallest of species in this study, Sanderling [53], while taking four or more months in the largest, Curlew [52]. Large species also tend to take more time to breed (e.g. 20.5–24 days in Turnstone versus 27–29 days in Eurasian Curlew *Numenius arquata* [54]). The longer duration of primary moult and longer breeding, together with lower fuel deposition rate [30], pose high time constraints for large species round the year, wherever they are, be it at their wintering grounds, migration and breeding grounds. We therefore interpret the shrinking of migration speed between seasons as that large species are time constrained and thus may adopt time-minimization strategy during both pre- and post-breeding migration.

Remarkably, however, instead of migrating at a similar speed across seasons, Curlew in fact showed a faster *partial* migration speed during post- compared to pre-breeding migration. This higher post-breeding migration speed can possibly be explained by potential differences in environmental conditions between the two seasons, such as food availability, day length [55] and weather conditions [56]. These differences in environmental conditions between the two seasons may not only affect Curlew but also other shorebird species, suggesting that despite faster migrations during pre-breeding migrations in the smaller species, conditions for migration might in fact be more favourable during post-breeding migration.

The main food for Curlews at their migratory staging sites consists of various crustacean species [57–60], whose density is higher during post- compared to pre-breeding migration along the EAAF ([61–63], but [64]). This overall higher food availability during post-breeding migration potentially enhances fuel deposition rate in Curlew, contributing to its higher post-breeding migration speed. Since Curlew and the other five studied species were tracked across 6 years, the observed seasonal difference in migration speed and the overall decline of migration speed with size is unlikely caused by the potential fact that species were coincidently tracked in years with particularly high seasonal difference in food availability.

Day length may also affect migration speed, where long days may result in higher net intake and thus fuel deposition rates as was suggested by Bauchinger U and Klaassen M [55] for passerines. Although most Scolopacidae, including Curlew, forage during both day and night [60], detecting prey by vision and tactile sensation [58, 65], their food intake might be more efficient during the day by using both detection methods while during the night vision detection might be limited. Indeed, Curlew experienced slightly longer days during post- compared to pre-breeding migration, potentially facilitating their relatively speedy migration during this season. Conversely, the smaller Sanderling and Turnstone, which migrated faster during the pre- compared to post-breeding migration, where in fact experiencing longer days during that season. Our data thus suggest that difference in day length during migration periods might to some extent have contributed to the seasonal difference in migration speed.

Another potentially important environmental factor in determining migration speed is weather conditions, and wind conditions in particular. Departing and flying with wind assistance enhances flying speed and saves energy and time during migration [56]. However, the only study investigating such potential differences between seasons at Chongming Dongtan in the south Yellow Sea (a stopover site along the EAAF), found that wind was on average supportive during both pre- and post-breeding migration in 3 years from 2007 to 2009 [66]. Although large variations existed within seasons, they did not find a significant difference in the strength of wind support between seasons. Therefore, weather was not considered to be the main driver of seasonal difference in migration speed observed in the present study. Rather, it implies that, as we predicted, large species were most likely under time selection pressure during not only pre- but also post-breeding migration. Future studies investigating seasonal difference in weather conditions along the migration route is warranted.

### Total migration distance

Similar total migration distances during pre- and post-breeding migration do not necessarily refute a differential migration strategy during the two seasons (i.e. time versus energy- minimization strategy, respectively). Still, longer migration distances during pre-breeding migration are considered to be the hallmark of time-minimization and considerably longer migration distances (up to 22%) have indeed been found in some migratory species during pre- compared to post-breeding migration [67]. In our study such seasonal difference was absent in all species but Tattler, despite that Turnstone and Great Knot were recorded to cover slightly longer distance during post-breeding migration, which fell within the range of geolocator errors [68]. Tattlers travelled a significantly shorter distance (10%) during post-breeding migration. All three Tattlers migrated via a more direct, easterly route when crossing the Pacific Ocean during their post-breeding migration, instead of stopping at more westerly islands such as the Philippines as shown during their pre-breeding migration [69].

Seasonal difference in total migration distance has been shown in some species along other flyways [67, 70]. The absence of any major seasonal difference in total migration distance might also be related to the geography of the EAAF. The most direct route traveling between the wintering grounds in New Zealand and Australia and the breeding grounds in the Siberian sub-Arctic and Arctic would involve a trans-Pacific crossing. This energetically demanding route option is avoided by most species under study but Bar-tailed Godwit [71, 72]. The next most straightforward route involves migrating using the coasts of China, Japan and/or Korea. Coincidently this route importantly runs through a region assumed to be one of the world’s most food abundant staging sites for shorebirds [73], with the Yellow Sea as one of its main areas. Not surprisingly, most shorebird species migrate via these staging sites during both seasons, resulting in similar total migration distance.

### Maximum step length

Longer maximum step length during pre- compared to post-breeding migration was recorded in Sanderling, Turnstone and Curlew. The longest steps occurred when the shorebirds under study crossed the Pacific Ocean from their wintering grounds in Australia to their staging sites on the east coast of China, spanning between 4400 and 9000 km. This very long jump during pre-breeding migration conforms to the prediction of a time-minimization strategy [43]. Instead of conducting a similar jump which could take them across the western Pacific when migrating back to the wintering grounds, most individuals divided this section of the journey into several small steps by stopping at islands in the Pacific Ocean, such as Java, Malaysia, Brunei and the Philippines, consistent with an energy-minimization strategy. Although apparently engaging in a time-minimization strategy during both pre- and post-breeding migration as judged from their migration speeds during both seasons, Curlew also made smaller steps during post-breeding than pre-breeding migration. However, the difference in maximum step length between the two seasons in Curlew (14%) was smaller than in Sanderling (25%) and Turnstone (25%).

## Conclusions

Although the optimal migration theory proposes that time-minimization is more relevant during pre- compared to post-breeding migration, the role of body size in comparing the two seasons has never been considered previously. The data presented to some extent support our initial hypothesis that large sized species are more time-constrained and thus tend to use a time-minimization strategy during both pre- and post-breeding migration.

Migration is a seriously threatened natural phenomenon and notably so along the EAAF, with many migrants being particularly impacted by habitat deterioration and loss in a major stopover region, the Yellow Sea [74]. In the face of these threats, identifying size and other species-specific constraints in their migratory behaviour and capacities may be of crucial importance in understanding and in assisting developing optimal conservation strategies to mitigate the threats of habitat deterioration. Considering the generally higher time pressure during pre- compared to post-breeding migration, the conservation of crucial sites for pre-breeding migratory preparation in shorebirds along the EAAF, such as the wintering grounds and important staging areas such as the Yellow Sea, are of profound significance to ensure a timely arrival at the breeding grounds. Furthermore, relatively large birds, which face high time constraints during both pre- and post-breeding migration, may be less flexible still in time and site use. Conservation of their habitat during both seasons is thus crucial for averting further population declines. The recent ongoing population declines in Eastern Curlew may be the hallmark of this [75]. However, size clearly is not the only crucial variable determining conservation needs since smaller shorebirds species along the EAAF, such as Curlew Sandpiper (*Calidris ferruginea*), may show similar dramatic declines [75].

## Additional files

Additional file 1: Table S1. Original data of seasonal difference for all four investigated migration variables for each individual. (DOCX 33 kb)

Additional file 2: Figure S1. Relationships between partial migration speed and lean body mass (g, log10) for six sandpiper species migrating northward along the East Asian-Australasian Flyway towards their breeding grounds. (DOCX 64 kb)
