# Supplementary material for: Time versus energy minimization migration strategy varies with body size and season in long-distance migratory shorebirds
Source: Mov Ecol. 2017 Nov 7;5:23. doi: 10.1186/s40462-017-0114-0 (PMC5674797; doi:10.1186/s40462-017-0114-0)
Supplement: Supplementary file 1 — Original data of seasonal difference for all four investigated migration variables for each individual. (DOCX 33 kb) [file 40462_2017_114_MOESM1_ESM.docx]

**Table S1**. Original data of seasonal difference (pre- minus post-breeding migration) for all four investigated migration variables for each individual across six sandpiper species migrating along the [East Asian-Australasian Flyway](http://www.eaaflyway.net/) between the Australia/New Zealand wintering grounds and the north eastern China/Russia Arctic breeding grounds. For some individuals migrating during equinox, their date arrival at a stopover site cannot be determined and thus for them *partial* migration speed cannot be estimated.

| **Species** | **Migration speed (km/d)** | | **Number of**  **staging sites** | **Total migration**  **distance (km)** | **Maximum**  **step length (km)** |
| --- | --- | --- | --- | --- | --- |
|  | ***Traditional*** | ***Partial*** |  |  |  |
| Sanderling | 295-200=95 | 213-162=51 | 5-6=-1 | 14170-13633=537 | 4801-4296=505 |
| Sanderling | 349-142=206 | 258-114=144 | 4-6=-2 | 14290-13240=1051 | 4737-3363=1374 |
| Sanderling | 405-233=172 | 236-209=27 | 4-4=0 | 14174-14689=-515 | 7094-7255=-160 |
| Sanderling | 328-151=177 | 195-133=62 | 4-8=-4 | 13108-13900=-792 | 7077-5301=1776 |
| Sanderling | 364-201=164 | 213-169=44 | 4-6=-2 | 14205-14841=-636 | 7179-2827=4352 |
| Sanderling | 312-140=171 | 226-123=103 | 5-6=-1 | 14021-14021=0 | 5203-5232=-29 |
| Sanderling | 327-149=179 | 207-129=79 | 3-7=-4 | 12768-14710=-1942 | 7166-4316=2850 |
| Sanderling | 370-231=139 | 273-194=79 | 4-5=-1 | 14432-14535=-103 | 4860-4148=712 |
| Sanderling | 293-155=138 | 148-129=19 | 3-5=-2 | 12592-14997=-2405 | 7107-6478=629 |
| Sanderling | 317-157=160 | 178-138=40 | 4-5=-1 | 13960-15088=-1127 | 7010-5242=1768 |
| Sanderling | 359-139=220 | 210-114=95 | 4-7=-3 | 14008-13750=258 | 7094-2785=4310 |
| Sanderling | 374-125=249 | 252-105=147 | 4-6=-2 | 14193-14232=-39 | 6382-2887=3495 |
| Ruddy Turnstone | 329-107=223 | 170-88=82 | 3-4=-1 | 13168-14699=-1531 | 6161-6894=-734 |
| Ruddy Turnstone | 306-188=118 | 184-143=41 | 4-5=-1 | 12552-14856=-2304 | 5562-4763=799 |
| Ruddy Turnstone | 259-178=81 | 244-129=115 | 5-4=1 | 14489-14074=415 | 4271-4929=-658 |
| Ruddy Turnstone | 285-200=85 | 153-168=-15 | 3-6=-3 | 12805-13390=-585 | 4394-2797=1597 |
| Ruddy Turnstone | 283-198=85 | 203-154=49 | 6-4=2 | 13299-14249=-950 | 4388-5083=-695 |
| Ruddy Turnstone | 289-182=107 | 212-135=77 | 4-4=0 | 13316-12937=378 | 4618-6248=-1630 |
| Ruddy Turnstone | 372-133=238 | 214-110=103 | 3-4=-1 | 13007-13466=-459 | 6439-6999=-559 |
| Ruddy Turnstone | 242-252=-10 | 112-204=-92 | 3-3=0 | 13316-13588=-272 | 7730-7027=703 |
| Ruddy Turnstone | 283-188=95 | 144-132=12 | 3-3=0 | 13281-13342=-61 | 4930-5259=-329 |
| Ruddy Turnstone | 273-170=103 | 139-141=-2 | 3-4=-1 | 13646-12926=720 | 7525-6360=1165 |
| Ruddy Turnstone | 330-108=222 | 164-74=91 | 2-5=-3 | 12865-13547=-682 | 7935-4478=3457 |
| Ruddy Turnstone | 330-174=157 | 170-127=43 | 2-7=-5 | 13544-14414=-871 | 7076-5246=1830 |
| Ruddy Turnstone | 360-128=233 | 216-92=124 | 3-4=-1 | 13329-13521=-193 | 7277-5049=2228 |
| Ruddy Turnstone | 371-103=268 | 210-81=129 | 3-4=-1 | 14114-14579=-464 | 7613-5263=2350 |
| Ruddy Turnstone | 265-210=56 | 129-177=-48 | 3-4=-1 | 13274-13828=-554 | 6119-5684=435 |
| Ruddy Turnstone | 364-179=185 | 143-134=9 | 2-4=-2 | 13108-13796=-689 | 8831-3789=5042 |
| Ruddy Turnstone | 322-185=137 | 169-162=6 | 2-5=-3 | 13191-13315=-124 | 7125-3552=3573 |
| Ruddy Turnstone | 405-193=212 | 229-152=77 | 4-4=0 | 13370-12964=406 | 4197-3378=819 |
| Ruddy Turnstone | 375-222=153 | 240-151=89 | 4-3=1 | 13131-12867=264 | 5039-4304=735 |
| Ruddy Turnstone | 355-211=144 | 184-190=-6 | 2-5=-3 | 13151-14781=-1629 | 5451-6938=-1487 |
| Ruddy Turnstone | 312-177=135 | 174-137=38 | 3-4=-1 | 12781-12715=67 | 5504-3368=2135 |
| Ruddy Turnstone | 398-176=221 | 287-118=169 | 4-3=1 | 13920-12872=1047 | 5586-3737=1849 |
| Ruddy Turnstone | 299-252=46 | 147-263=-116 | 3-4=-1 | 13138-13372=-234 | 5617-6757=-1141 |
| Ruddy Turnstone | 381-211=170 | 214-174=40 | 3-3=0 | 13721-12670=1052 | 7073-3743=3329 |
| Ruddy Turnstone | 364-124=240 | 242-93=149 | 4-5=-1 | 12745-13039=-294 | 4289-3218=1071 |
| Ruddy Turnstone | 396-201=195 | 213-152=61 | 3-4=-1 | 13056-14464=-1408 | 3987-4195=-207 |
| Ruddy Turnstone | 330-199=131 | 174-169=5 | 3-4=-1 | 12873-14315=-1443 | 7119-6107=1012 |
| Ruddy Turnstone | 418-297=121 | 238-229=9 | 3-4=-1 | 12956-13650=-693 | 4905-5265=-360 |
| Ruddy Turnstone | 313-269=44 | 149-217=-69 | 3-5=-2 | 13141-13199=-58 | 7788-4461=3327 |
| Ruddy Turnstone | 258-134=124 | 156-94=62 | 3-4=-1 | 14209-13298=911 | 7365-3506=3859 |
| Ruddy Turnstone | 264-247=18 | 121-215=-94 | 2-5=-3 | 12938-13066=-128 | 7750-4299=3451 |
| Ruddy Turnstone | 257-215=42 | 130-181=-51 | 3-4=-1 | 13387-13762=-375 | 7396-4348=3048 |
| Ruddy Turnstone | 260-278=-18 | 128-220=-92 | 3-4=-1 | 13017-13069=-51 | 5851-3492=2359 |
| Ruddy Turnstone | 283-181=102 | 142-131=11 | 3-4=-1 | 13580-13594=-14 | 5011-3754=1257 |
| Ruddy Turnstone | 375-213=162 | 198-172=27 | 3-5=-2 | 13488-13398=90 | 7534-3259=4275 |
| Ruddy Turnstone | 263-182=80 | 154-126=28 | 3-3=0 | 13141-13128=14 | 6350-4812=1538 |
| Ruddy Turnstone | 277-155=122 | 144-126=18 | 3-4=-1 | 13564-13752=-188 | 7498-6607=891 |
| Ruddy Turnstone | 253-224=28 | 124-167=-44 | 4-3=1 | 13392-12796=596 | 4411-6787=-2376 |
| Ruddy Turnstone | 224-186=39 | 114-131=-18 | 3-3=0 | 13239-13004=236 | 7223-4664=2559 |
| Ruddy Turnstone | 256-160=96 | 256-114=142 | 4-5=-1 | 13580-14088=-508 | 7214-3428=3786 |
| Ruddy Turnstone | 353-154=200 | 236-93=144 | 3-4=-1 | 13427-13381=45 | 5669-3962=1708 |
| Ruddy Turnstone | 275-234=42 | 165-176=-11 | 4-5=-1 | 13483-13779=-296 | 5876-4202=1675 |
| Ruddy Turnstone | 238-129=109 | 146-98=48 | 5-4=1 | 13087-14278=-1191 | 5500-7288=-1788 |
| Ruddy Turnstone | 304-193=111 | 163-136=27 | 3-3=0 | 13378-13290=88 | 5611-4256=1355 |
| Ruddy Turnstone | 243-213=29 | 122-156=-34 | 5-5=0 | 13584-13022=563 | 5680-2247=3432 |
| Ruddy Turnstone | 329-215=113 | 199-136=63 | 4-3=1 | 13141-13120=20 | 6558-3706=2852 |
| Ruddy Turnstone | 321-284=37 | 196-238=-42 | 3-4=-1 | 14120-13609=511 | 6860-4217=2642 |
| Ruddy Turnstone | 286-203=83 | 151-148=3 | 4-4=0 | 13166-13604=-438 | 5422-3488=1934 |
| Ruddy Turnstone | 329-219=110 | 182-155=26 | 3-5=-2 | 14130-13776=354 | 7227-4325=2903 |
| Ruddy Turnstone | 281-208=73 | 144-153=-9 | 3-4=-1 | 13466-13500=-34 | 7550-5472=2078 |
| Ruddy Turnstone | 301-189=112 | 155-143=12 | 3-4=-1 | 13238-14571=-1333 | 7491-3654=3837 |
| Ruddy Turnstone | 377-183=194 | 209-131=78 | 3-4=-1 | 13196-13716=-520 | 7127-4536=2591 |
| Ruddy Turnstone | 315-159=156 | 160-113=47 | 3-4=-1 | 13241-13395=-154 | 7626-2955=4671 |
| Ruddy Turnstone | 258-364=-106 | 120-275=-155 | 4-2=2 | 13441-13120=321 | 7676-8795=-1119 |
| Ruddy Turnstone | 338-293=46 | 168-232=-64 | 3-3=0 | 13200-13170=30 | 7994-8146=-151 |
| Ruddy Turnstone | 283-227=56 | 136-187=-51 | 3-3=0 | 13303-13169=134 | 7604-5999=1604 |
| Ruddy Turnstone | 282-162=120 | 136-136=0 | 3-5=-2 | 13252-13624=-372 | 7670-4325=3345 |
| Ruddy Turnstone | 328-226=102 | 249-193=56 | 4-5=-1 | 13448-13763=-314 | 3997-5834=-1837 |
| Ruddy Turnstone | 265-163=102 | 126-119=7 | 2-3=-1 | 13228-13182=46 | 7686-7323=363 |
| Ruddy Turnstone | 317-148=169 | 228-106=123 | 3-3=0 | 13946-13439=507 | 5051-4322=729 |
| Grey-tailed Tattler | 333-285=49 | 214-192=23 | 3-3=0 | 11665-10816=849 | 5445-4732=714 |
| Grey-tailed Tattler | 329-361=-32 | - | 2-1=1 | 10868-9758=1110 | 6954-9758=-2804 |
| Grey-tailed Tattler | 373-148=225 | 170-135=35 | 2-3=-1 | 10816-9757=1059 | 6902-6348=554 |
| Red Knot | 254-158=96 | 91-150=-59 | 3-6=-3 | 14742-15325=-584 | 10112-6612=3500 |
| Red Knot | 183-164=19 | 179-132=47 | 3-5=-2 | 10988-11163=-175 | 6333-4730=1603 |
| Great Knot | 341-171=170 | 247-163=84 | 3-4=-1 | 10234-10789=-555 | 4312-6251=-1939 |
| Great Knot | 191-222=-32 | 147-184=-37 | 3-4=-1 | 9541-9786=-245 | 4438-4602=-164 |
| Great Knot | 184-146=38 | 144-124=20 | 4-4=0 | 9755-10057=-302 | 3489-4568=-1079 |
| Great Knot | 171-212=-41 | 128-183=-55 | 3-3=0 | 9729-9968=-239 | 3500-6203=-2703 |
| Great Knot | 339-180=159 | 196-174=21 | 3-4=-1 | 9162-9533=-371 | 4655-3169=1486 |
| Great Knot | 177-186=-9 | 138-182=-44 | 3-3=0 | 9712-10024=-311 | 3911-4681=-771 |
| Great Knot | 180-212=-32 | 136-183=-47 | 4-3=1 | 9557-10187=-630 | 3248-6159=-2911 |
| Far Eastern Curlew | 278-168=110 | 44-149=-105 | 2-3=-1 | 9744-10275=-531 | 8556-7039=1517 |
| Far Eastern Curlew | 229-181=49 | 30-162=-132 | 2-3=-1 | 9404-10110=-706 | 8377-7039=1338 |
| Far Eastern Curlew | 308-181=127 | - | 2-3=-1 | 9852-10656=-803 | 8477-7287=1190 |
| Far Eastern Curlew | 273-134=138 | 32-103=-71 | 2-2=0 | 9820-9813=7 | 8891-7491=1400 |
| Far Eastern Curlew | 215-118=97 | 57-95=-37 | 2-3=-1 | 9675-10411=-735 | 7491-6118=1373 |
| Far Eastern Curlew | 340-162=178 | - | 2-2=0 | 9852-9852=0 | 8477-8477=0 |
| Far Eastern Curlew | 266-189=77 | - | 2-2=0 | 9852-9852=0 | 8477-8477=0 |
| Far Eastern Curlew | 244-157=87 | 47-148=-100 | 2-3=-1 | 9508-9593=-84 | 7997-7997=0 |
| Far Eastern Curlew | 177-146=31 | 48-121=-74 | 3-2=1 | 10423-9605=818 | 7997-7997=0 |
