# Supplementary material for: Time versus energy minimization migration strategy varies with body size and season in long-distance migratory shorebirds
Source: Mov Ecol. 2017 Nov 7;5:23. doi: 10.1186/s40462-017-0114-0 (PMC5674797; doi:10.1186/s40462-017-0114-0)
Supplement: Supplementary file 2 — Relationships between partial migration speed and lean body mass (g, log10) for six sandpiper species migrating northward along the East Asian-Australasian Flyway towards their breeding grounds. (DOCX 64 kb) [file 40462_2017_114_MOESM2_ESM.docx]

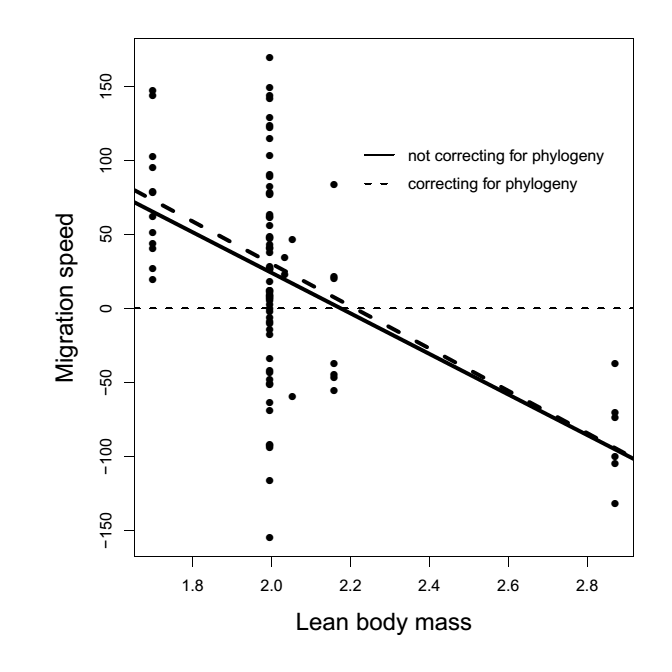


**Figure S1**. Relationships between partial migration speed and lean body mass (g, log10) for six sandpiper species migrating northward along the [East Asian-Australasian Flyway](http://www.eaaflyway.net/) towards their breeding grounds. Black solid and dashed lines indicate the slope not correcting and correcting for phylogeny, respectively.
